# Evaluation of Antileishmanial and Antibacterial Activity of Bioconjugate Guanidine–Temporin

**DOI:** 10.3390/ph19060835

**Published:** 2026-05-27

**Authors:** Gabriel Antunes Santoro, Natalia Caroline Souza Costa, Sarah Tolentino Rocha Brandão, Jhonatan Santos de Lima, Angela Maria Arenas Velasquez, Luana Ribeiro dos Anjos, Cauã Dias Abrão, João Victor Marcelino de Souza, Marcela Nunes Argentin, Ilana Lopes Baratella da Cunha Camargo, Marcia A. S. Graminha, Eduardo Rene P. Gonzalez, Eduardo Maffud Cilli

**Affiliations:** 1 Department of Biochemistry and Organic Chemistry, Institute of Chemistry, São Paulo State University (UNESP), Araraquara 14800-060, SP, Brazil; ga.santoro@unesp.br (G.A.S.); sarah.brandao@unesp.br (S.T.R.B.); joao-victor.souza@unesp.br (J.V.M.d.S.); 2 School of Pharmaceutical Sciences, São Paulo State University (UNESP), Araraquara 14800-903, SP, Brazil; natalia.costa@unesp.br (N.C.S.C.); jhonatan.lima@unesp.br (J.S.d.L.); a.velasquez@unesp.br (A.M.A.V.); marcia.graminha@unesp.br (M.A.S.G.); 3 School of Sciences and Technology, São Paulo State University (UNESP), Presidente Prudente 19060-080, SP, Brazil; luana.anjos@unesp.br (L.R.d.A.); dias.abrao@unesp.br (C.D.A.); 4 Department of Physics and Interdisciplinary Science, São Carlos Institute of Physics, University of São Paulo (USP), São Carlos 13563-120, SP, Brazil; marcela.argentin@usp.br (M.N.A.); ilanacamargo@ifsc.usp.br (I.L.B.d.C.C.)

**Keywords:** bioconjugate, antimicrobial peptide, bactericidal, antileishmanial, guanidine

## Abstract

**Background**: Peptides are promising tools in medicine for viral, bacterial, and parasitic infections. MAP1 and MAP2 are peptides with antimicrobial activity. Aiming to increase selectivity, enhance antimicrobial activity, and reduce its cytotoxicity, two bioconjugates (GVL1-MAP2 and GVL1-MAP1) with the guanidine derivative group in the N-terminus position were synthesized. **Methods**: The peptides and bioconjugates were synthesized by SPPS. The biological activity and inhibition of the CPB enzyme were evaluated. **Results**: Antileishmanial activity was evaluated, and the bioconjugates exhibited higher activity in both the promastigote and amastigote forms than isolated GVL1 and peptides alone. **Discussion**: GVL1-MAP2 bioconjugate demonstrated not only the greatest activity against *L. amazonensis* in the promastigote (IC_50_ = 3.2 μM) and amastigote (IC_50_ = 0.6 μM) forms but also prevented the parasite from infecting new host cells, reducing the infection rate by 3-fold compared to the untreated control. Similar results were obtained in *L. infantum*, with IC_50_ = 2.5 and 1.0 μM for promastigote and amastigote forms, respectively. In active serum, GVL1-MAP2 continued to show high activity. GVL1-MAP2 also showed bactericidal activity against most strains tested. The bioconjugate GVL1-MAP2 showed lower cytotoxicity than GVL1-MAP1 and amphotericin B in macrophages. Permeabilization studies and enzyme inhibition revealed that the peptide acts via at least two distinct mechanisms, with the primary mechanism targeting the membrane and inner targets as an inhibitor of the CPB enzyme. **Conclusions**: These data demonstrated that the synthesis of bioconjugates can be a tool for the development of bactericidal and antileishmanial compounds with improved potency and selectivity.

## 1. Introduction

Infectious diseases are caused by microorganisms such as bacteria, parasites, and fungi and represent a global challenge, especially in regions with precarious socioeconomic conditions, where access to adequate treatments is limited. These diseases represent a major public health concern, especially considering the increasing number of immunocompromised patients and those living in poverty [1].

Leishmaniasis is a tropical, parasitic infection caused by the protozoan *Leishmania* spp. and transmitted through the bite of an infected female Phlebotomine [2]. These are considered neglected diseases and occur in underdeveloped regions, such as the Global South. Despite leishmaniasis being an endemic disease, little to no effort is employed in treating and preventing it. Leishmaniasis remains a major global health threat, affecting millions of people primarily in developing countries. The clinical management of this neglected tropical disease is severely hampered by a limited therapeutic arsenal and the emergence of drug-resistant *Leishmania* strains. Resistance to pentavalent antimonials is now widespread in several endemic regions, and decreased susceptibility to second-line drugs, such as amphotericin B and miltefosine, has been increasingly reported. The use of extremely cytotoxic drugs such as amphotericin B (Amp B), which must be injected in the patient in several, painful shots, leads to severe side effects [3]. This alarming trend of drug resistance, coupled with the high toxicity and cost of current treatments, underscores an urgent need for the development of novel, safe, and effective antileishmanial agents, such as antimicrobial peptides and their bioconjugates [3].

Other infectious diseases, particularly bacterial ones, are also on the rise, with cases emerging worldwide and medical treatments that were once remarkably effective now becoming flawed and outdated.

The World Health Organization (WHO) has categorized the bacteria most urgently needed for new therapies as ESKAPE (*Enterococcus faecium*, *Staphylococcus aureus*, *Klebsiella pneumoniae*, *Acinetobacter baumannii*, *Pseudomonas aeruginosa*, and *Enterobacter* species). This classification was based on criteria such as mortality rate, treatability, healthcare burden, and resistance prevalence, among others (WHO, 2019) [4]. Antibiotic resistance makes treatment of these and other bacterial infections challenging, increasing associated morbidity and mortality. Prolonged and uncontrolled use of drugs against bacteria and parasites promotes the prevalence of resistant pathogens [5]. Furthermore, there are a few therapeutic options for treating bacteria. Therefore, the search for new therapeutic alternatives is particularly important.

Antimicrobial peptides (AMPs) are short amino acid chains that occur from mammals to fungi that serve as a defense mechanism against predators [6]. They have had a plethora of applications in modern medicine and research fields when fighting the recent downfall of antibiotic efficacy [5]. The use of AMPs has shown effects on bacterial and protozoan cell membranes, thus leading to the death of the pathogen through a vast number of mechanisms, such as cell penetration and pore-inducing, disturbing the lipid bilayer structure of the cell, thus inducing its death [7].

Our group has evaluated bioconjugates containing AMPs and organic active compounds and has obtained promising results against parasites, bacteria, and viruses [7,8]. Recently, naturally occurring molecules that contain a guanidine center (or moiety) have been studied. Guanidines are widely used due to their oxidative phosphorylation properties [9], which can target intracellular parts of the pathogen. The data supports the actions of guanidine-derivative compounds at the cellular level, inhibiting enzymes such as cysteine protease B (CPB) in the *Leishmania* spp. protozoan, an enzyme responsible for its thriving when phagocytosed by a macrophage and, therefore, infection of the host. Its inhibiting action favors the treatment of the disease, thus not allowing the parasite to infect the cell [7,10].

Bioconjugates containing guanidine derivatives and peptides have already been explored by our group’s research against antileishmanial activities [7,11] using a cell penetration peptide (CPP) and the TSHa peptide against *Leishmania*. Given the potential of AMPs and guanidine derivatives, we synthesized two bioconjugates containing guanidine derivatives with the peptide analogues HP-MAP1 (MAP1) and HP-MAP2 (MAP2) (amide form), derived from temporin-PTa. Previously, these peptides have shown antimicrobial activity [12], while temporin-PTa is antiparasitic. A guanidine derivative called GVL1 ((Z)-4-((2-benzoyl3-(4-bromophenyl)guanidino)methyl) benzoic acid) (Figure S1) was used for this conjugation. Thus, this work explores the use of new bioconjugates, GVL1-MAP1 and GVL1-MAP2, as promising molecules against some ESKAPE bacterial strains and for antileishmanial activity in the treatment of *Leishmania* spp.

## 2. Results

### 2.1. Synthesis of GVL1-MAP1 and GVL1-MAP2 Bioconjugates

**Figure 1:**
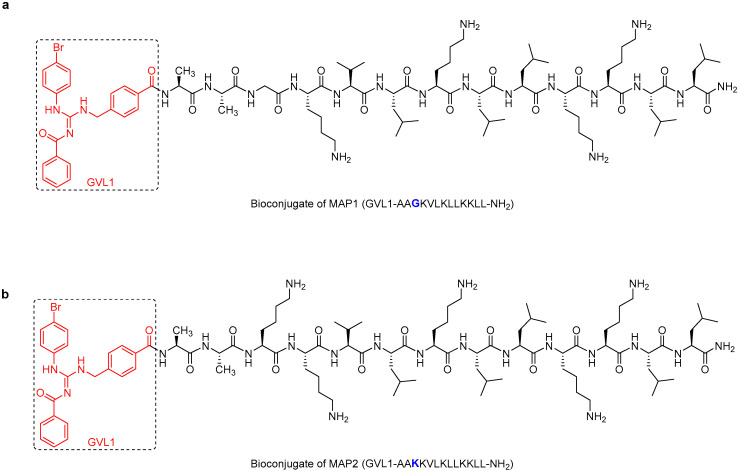
Chemical structures of bioconjugates GVL1-MAP1 (**a**) and GVL1-MAP2 (**b**). The guanidine substituent GVL1 is inside the dashed box and highlighted in red. MAP1 and MAP2 peptides are displayed in black. MAP1 and MAP2 bioconjugates differ by one amino acid (highlighted in blue).

**Figure 2:**
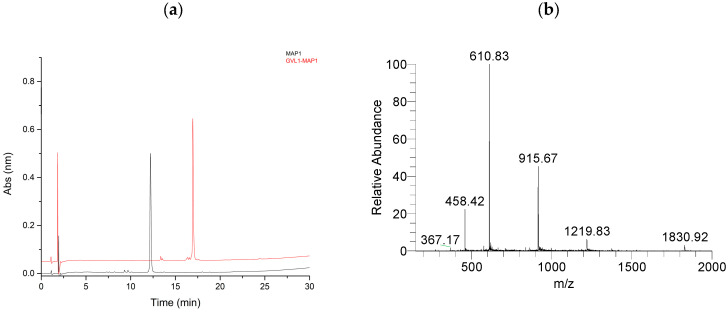
HPLC profile of the pure MAP1 and GVL1-MAP1 compounds (**a**) and mass spectrum (**b**) of the pure GVL1-MAP1 bioconjugate. Theoretical MW = 1826.9 g/mol. MW/Z ratio = 914.5, 610.0, and 457.7 for Z = 2, 3, and 4, respectively. Experimental MW/Z ratio = 915.7, 610.8 and 458.4 for Z = 2, 3, and 4, respectively.

**Figure 3:**
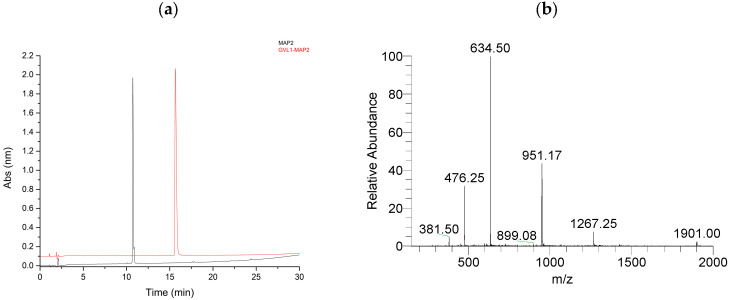
HPLC profile of the pure MAP2 and GVL1-MAP2 compounds (**a**) and mass spectrum (**b**) of the pure GVL1-MAP2 bioconjugate. Theoretical MW = 1898.0 g/mol. MW/Z ratio = 950.0, 633.7, and 475.5 for Z = 2, 3, and 4, respectively. Experimental MW/Z ratio = 951.2, 634.5, and 476.3 for Z = 2, 3, and 4, respectively.

The MAP1 (AAGKVLKLLKKLL-NH_2_) (Figure S2) and MAP2 (AAKKVLKLLKKLL-NH_2_) (Figure S3) peptides were synthesized via SPPS (Solid Phase Peptide Synthesis) [13] using the Fmoc protocol. GVL1 was coupled to the N-terminal group of MAP1 (Figure 1a) and MAP2 (Figure 1b) using HATU/DIEA via its carboxyl group [7]. The peptides and bioconjugates were purified by reverse-phase HPLC in semi-preparative mode (Figure 2a and Figure 3a). The material obtained was analyzed by mass spectrometry, confirming that the desired material had been obtained (Figure 2b and Figure 3b). The mass spectra of both MAP1 and MAP2 peptides are in the Supplementary Data (Figures S4 and S5).

### 2.2. Biological Assays

#### 2.2.1. Antileishmanial and Cytotoxic Activities Evaluation

The antileishmanial activity against *L. amazonensis* showed that GVL1 had IC_50_ values of 51.8 and 7.5 μM for the promastigote and amastigote forms, respectively, and the MAP1 peptide showed IC_50_ values of 43 μM in promastigote and 10 μM in amastigote forms (Table 1). The GVL1-MAP1 bioconjugate exhibited higher activity than GVL1 or MAP1 alone in both the promastigote and amastigote forms, with IC_50_ values of 7.3 and 0.9 μM, respectively (Table 1). In amastigotes, the increase in activity was approximately 8.5 times greater than that of GVL1 alone and 11 times greater than that of the MAP1 peptide. The GVL1-MAP1 bioconjugate showed CC_50_ = 21 μM in murine peritoneal macrophages, a lower value when compared to the MAP1 peptide alone and, similarly, Amp B. The selectivity index (SI) for this compound was smaller than that of Amp B, indicating worse biological properties. The SI indicates the compound’s toxicity towards the parasite relative to host mammalian cells (SI = CC_50_/IC_50_). Regarding the GVL1-MAP2 bioconjugate, it showed the best antileishmanial activity for the promastigote (IC_50_ = 3.2 µM) and amastigote (IC_50_ = 0.6 µM) forms (Table 1), with an increase in potency of approximately 2-fold when compared to GVL1-MAP1. In addition, this bioconjugate has the highest CC_50_ value (125 µM) and selectivity index (SI = 201). This last value was approximately six times higher than that of Amp B (SI = 32) and seven times higher than that of the MAP2 peptide, demonstrating that the coupling of GVL1 increases the potency and selectivity of GVL1 and MAP2 alone.

The bioconjugates with better antileishmanial activity against *L. amazonensis*, GVL1-MAP2, and the MAP2 peptide were also evaluated against *L. infantum*. The results were similar to those obtained for *L. amazonensis.* GVL1-MAP2 showed better potency and SI, with an IC_50_ value of 1.0 µM for the amastigote form, similar to that of the control drug Amp B (1.25 µM) (Table 2). In addition, this compound was more selective for the parasite, with SI = 125, higher than that obtained for Amp B (SI = 19), indicating that the bioconjugate was 5.2 times more selective for the parasite than Amp B.

**Figure 4:**
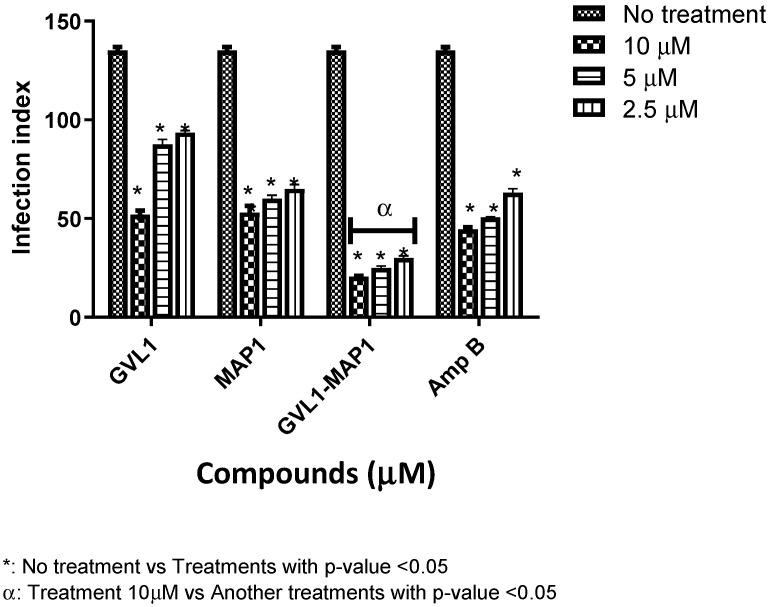
Infection indices for GVL1, MAP1, GVL1-MAP1, and Amp B in intracellular amastigotes of *L. amazonensis*. The infection index was calculated after 24 h of treatment with each compound. The negative control was untreated *L. amazonensis* intracellular amastigotes. The data are expressed as the mean and standard deviation (SD) for three independent experiments. Two-way ANOVA (*p* < 0.05) was applied, where “*” indicates a significant difference between the tested compounds and the untreated control, and “α” indicates a significant difference between the tested concentrations of each compound.

The ability to infect new macrophages was evaluated at concentration values of 2.5, 5.0 and 10 μM. The lower the infection rate of *Leishmania*, the greater its ability to prevent the infection of new macrophages. GVL1-MAP1 in *L. amazonensis,* after 24 h, decreased the infection rate almost 6.7-fold in the three tested concentrations in comparison to infected cells that were not treated (Figure 4). In comparison to the control drug Amp B, the bioconjugate was 3.5 times more effective at 10 μM, while the MAP1 peptide and GVL1 alone had smaller infection index values than GVL1-MAP1.

**Figure 5:**
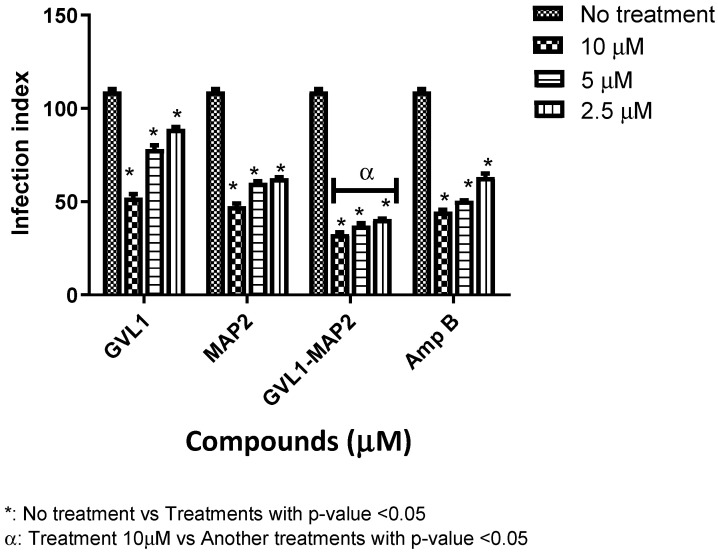
Infection indices for GVL1, MAP 2, GVL1-MAP 2, and Amp B in intracellular amastigotes of *L. amazonensis*. The infection index was calculated after 24 h of treatment with each compound. The negative control was untreated *L. amazonensis* intracellular amastigotes. The data are expressed as the mean and standard deviation (SD) for three independent experiments. Two-way ANOVA (*p* < 0.05) was applied, where “*” indicates a significant difference between the tested compounds and the untreated control, and “α” indicates a significant difference between the tested concentrations of each compound.

The GVL1-MAP2 bioconjugate also showed greater selectivity for the parasite than for the mammalian host cell. Moreover, it was also able to prevent the parasite from infecting new host cells. There was a 3-fold decrease in the infection rate when compared to the untreated control for *L. amazonensis* (Figure 5), and the infection index value was also lower than that found in relation to Amp B. The lower infection rate found for GVL1-MAP1 could be related to its higher toxicity in macrophages.

**Figure 6:**
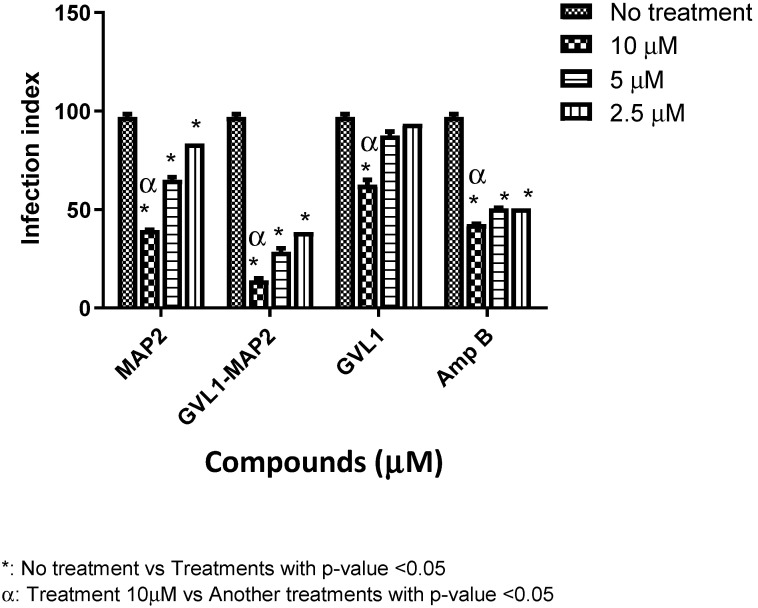
Infection indices for GVL 1, MAP 2, GVL 1-MAP 2, and Amp B in intracellular amastigotes of *L. infantum.* The infection index was calculated after 24 h of treatment with each compound. The negative control was untreated *L. infantum* intracellular amastigotes. The data are expressed as the mean and standard deviation (SD) of three independent experiments. Two-way ANOVA (*p* < 0.05) was applied, where “*” indicates a significant difference between the tested compounds and the untreated control, and “α” indicates a significant difference between the tested concentrations of each compound.

The bioconjugate GVL1-MAP2 was also able to reduce the infection rate for in vitro treatment with *L. infantum*; compared to macrophages infected without treatment, there was a 7.5-fold decrease in the infection rate (Figure 6) at 10 μM. In comparison to the control drug Amp B, there was a 3-fold decrease (Figure 6), demonstrating that, in addition to improving the selectivity of this compound, it is also able to interfere with the infection process, preventing new macrophages from being infected.

#### 2.2.2. Stability Assay with Active Fetal Bovine Serum

In inactivated serum, the MAP2 peptide alone had not shown activity against *Leishmania* in the promastigote form, but in the presence of active serum, the IC_50_ values were 81.5 and 17.4 μM for *L. amazonensis* and *L. infantum*, respectively (Table 3).

The bioconjugate GVL1-MAP2 exhibited IC_50_ = 9.9 μM against *L. amazonensis* promastigote and 4.4 μM against *L. infantum* (Table 3). Despite undergoing partial degradation, the bioconjugate maintained significant biological potency. The decrease in activity (IC_50_) was 3- and 2-fold compared with those found for *L. amazonensis* and for *L. infantum*, respectively, in inactive serum.

#### 2.2.3. Antibacterial Assays

The antibacterial activity of the bioconjugates was tested against Gram-positive and Gram-negative bacteria (*Enterococcus faecium* (Gram-positive); *Staphylococcus aureus* (Gram-positive); *Klebsiella pneumoniae* (Gram-negative); *Acinetobacter baumannii* (Gram-negative); *Pseudomonas aeruginosa* (Gram-negative); *Enterobacter* spp. (Gram-negative)), including ESKAPE bacteria, and expressed as minimum inhibitory concentration (MIC) and minimum bactericidal concentration (MBC) (Table 4 and Table 5). The *Staphylococcus epidermidis* is not classified within the ESKAPE group but was included in this study due to its status as a major opportunistic pathogen in hospital settings. The *S. epidermidis* strain is a well-characterized biofilm producer, representing a significant challenge in the treatment of device-related infections and chronic wounds, exhibiting multidrug resistance profiles similar to those found in ESKAPE species [5]. Except for *S. epidermidis* ATCC 35984, the GVL1-MAP1 bioconjugate was more active against Gram-positive bacteria than MAP1 (Table 4), particularly against the vancomycin-resistant *E. faecium* ATCC 700221 (MIC = 8 mg/mL). For Gram-negative bacteria, the GVL1-MAP1 bioconjugate showed activity only against *A. baumannii* ATCC 19606 and *P. aeruginosa* ATCC 27853, and the MIC values were higher than those of the MAP1 peptide. GVL1-MAP2 demonstrated activity against all tested Gram-positive bacteria, with higher activity than the isolated peptide MAP2, except for the strain *S. epidermidis* ATCC 35984. For Gram-negative bacteria, GVL1-MAP2 showed activity against all strains tested, but it was more active than MAP2 alone only in *P. aeruginosa* ATCC 27853 (Table 5).

### 2.3. Mechanism of Action

#### 2.3.1. Vesicle Permeabilization

**Figure 7:**
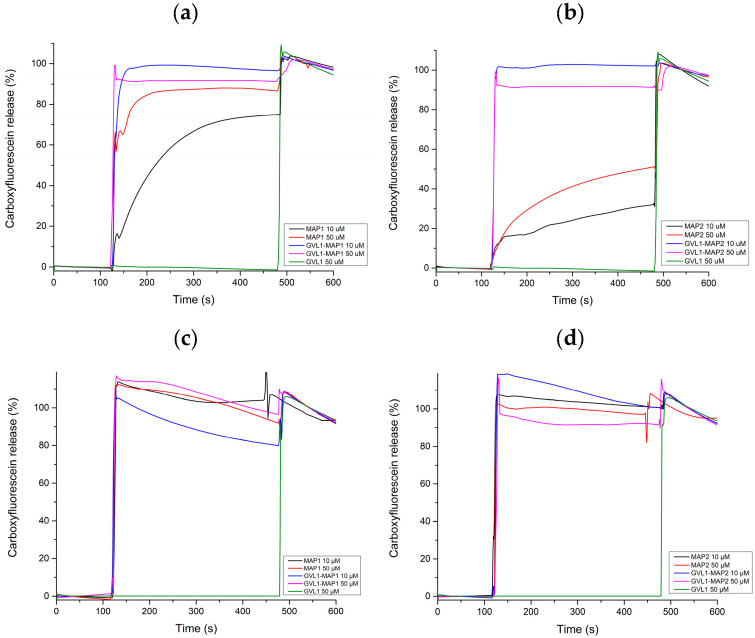
Fluorescein release profile of the vesicles mimicking a eukaryotic membrane containing 1-POPC and cholesterol (**a**,**b**) or POPC, POPS and ergosterol (**c**,**d**) in the presence of the compounds GVL1 (50 μM) and MAP1, GVL1-MAP1, MAP2 and GVL1-MAP2 (10 μM and 50 μM).

To evaluate a possible mechanism of action of the GVL1-MAP2 bioconjugate and the MAP2 peptide on the membranes, a permeabilization assay based on increasing the fluorescence of carboxyfluorescein (CF) leakage promoted by pore formation in unilamellar vesicles [15] was used. Thus, two membrane mimetic systems were evaluated, one containing 1-palmitoyl-2-oleoyl-sn-glycero-3-phosphocholine (POPC), 1-palmitoyl-2-oleoyl-sn-glycero-3-phospho-L-serine (POPS), and ergosterol, as a *Leishmania* membrane mimetic, and the other containing 1-palmitoyl-2-oleoyl-sn-glycero-3-phosphocholine (POPC) and cholesterol, as a eukaryotic membrane mimetic [16,17,18]. GVL1 alone did not permeabilize the vesicle mimetics of either mimetic vesicle (Figure 7), indicating, as expected, that the mechanism of action of GLV1 is not related to the cell membrane. The bioconjugates were able to cause faster membrane damage than peptides in eukaryotic mimetic membrane-containing POPC/cholesterol (Figure 7a,b), indicating that the conjugation strategy increases the capacity for membrane damage. These data are in accordance with the CC_50_ found in macrophages. The CC_50_ values of the bioconjugates were smaller than those found for the peptides. The peptides alone or conjugated with GVL1 in the *Leishmania* membrane mimetic (Figure 7c,d) caused rapid and more intense membrane damage than in POPC/cholesterol vesicles.

#### 2.3.2. Cysteine Protease Enzyme Inhibition

We evaluated the action of MAP2 and GVL1-MAP2 on recombinant cysteine protease from *Leishmania mexicana* (CPB) (rCPB2.8ΔCTE), an important enzyme for the cell viability of the parasite. The peptide and bioconjugate inhibited enzymatic activity by approximately 95% and 89%, respectively, at 20 μM (Table 6). The IC_50_ for CPB inhibitory activity was also determined, demonstrating the peptide’s superior inhibitory activity against CPB.

## 3. Discussion

The retention times of the bioconjugates in the HPLC profiles were higher than those of peptides, indicating an increase in the hydrophobicity of the compound. These data are in agreement with the low solubility of the guanidine derivatives. The peptide increases the solubility of the guanidine derivative and its activity and decreases the toxicity.

One focus of this work is the development of molecules with anti-*Leishmania* activity. *L. amazonensis* is one of the species that causes cutaneous leishmaniasis (CL) and affects 12 million people, with 2 million new cases occurring annually [19]. Therefore, the development of less toxic and less painful therapies is needed [20]. However, the financial resources are limited for developing new molecules, vaccines, and diagnostic methods for leishmaniosis, a disease that mainly affects underprivileged people [21].

Peptides from the temporin class have the membrane as the main mechanism of action [22,23] and could facilitate the entry of active compounds, such as guanidine derivatives, into cells and promote inhibitory activity in internal proteins [12]. Even though temporin-class peptides are toxic to mammalian cells, this problem could be resolved by modifying the peptide sequence [12,24]. A study involving the peptide TSHa demonstrated that modifications of the peptide sequence at positions 4 and 7 (2 Gly for 2 Ala) reduced the toxicity of these analogues to red blood cells and other cell lines. This occurred due to an increase in hydrophobicity [25]. MAP1 and MAP2 were derived from temporin-PTa (FFGSVLKLIPKIL). MAP1 has the amino acids Ser^4^ and Pro^10^ replaced by Lys. In MAP2, Gly^3^ was replaced by a Lys. In both peptides, alanine residues replaced Phe at the N-terminus to assist the extension of the α-helical segment, and isoleucine residues were replaced by leucine residues aiming at reduced hydrophobicity. Both analogues displayed in vitro activity against bacteria without presenting hemolytic effects [12].

In this work, MAP2 showed a higher CC_50_ than MAP1 in macrophages. These results were attributed to the higher hydrophobicity of MAP1 and the different α-helical amphipathic structure content [12]. The α-helical amphipathic structure content was also related to a reduction in cytotoxicity activity in mammalian cells [26]. The antileishmanial activity of MAP1 was higher than that of MAP2 in promastigote form, but it was lower in amastigote form. The *Leishmania* membrane undergoes radical remodeling to adapt to its host environments. Promastigotes possess a glycocalyx dominated by lipophosphoglycan (LPG), which imparts a strong negative surface charge necessary for sandfly midgut attachment [27]. In terms of lipid composition, they are rich in ergosterol-type sterols and unsaturated fatty acids, maintaining high membrane fluidity. Conversely, amastigotes drastically downregulate LPG, replacing it with smaller glycoinositolphospholipids (GIPLs) and sphingolipids (such as inositol phosphorylceramide) and cholesterol scavenged from the host. These modifications result in a neutralized or less negative charge and a more rigid membrane in amastigote form [28,29]. The difference in the membrane composition could be related to different anti-*Leishmania* activities in the amastigote and promastigote forms.

The use of bioconjugates containing membrane-bound peptides can facilitate the entry of the guanidine derivative into the cell and promote its inhibitory activity on intracellular proteins via transient pore formation [30]. In the amastigote form, bioconjugation increased the IC_50_ by approximately 10 times compared to GVL1, and the values were similar to those of Amp B. Moreover, the SI of GVL1-MAP2 was 6.3 and 6.6 times higher than that of Amp B for *L. amazonensis* and *L. infantum*, respectively. The addition of the guanidine derivative to the N-terminus of the peptides decreased the CC_50_. This could be due to the charge of the N-terminus group. The bioconjugation of GVL1 in the amino-terminal region decreased the overall charge of the peptide, which could make the initial interaction with mammalian cell membranes difficult, causing a decrease in the CC_50_ value. The N-terminus group is important to the biological activity of the peptides and could change the selectivity of the bioconjugates [31]. This data substantiates the exceptional biological activity of the bioconjugate.

The synthesis of the bioconjugate may also have other effects on the peptide structure. The use of AMPs in clinical applications remains a challenge due to stability and bioavailability [32]. Several strategies have been employed to increase the stability of peptides, including the incorporation of non-natural amino acids, modification to the N- and/or C-terminal, cyclization, the use of non-peptide structures (peptidomimetics), and multimerization [33]. To evaluate the influence of the GVL1 compound on the stability of MAP2 (GVL1-MAP2), the antileishmanial activity was performed in the presence of active fetal bovine serum added to the culture medium of the *L. amazonensis* and *L. infantum* parasites in their promastigote form. The active fetal bovine serum contains proteases that can degrade these molecules [34]. The bioconjugates showed degradation but sustained relevant biological activity. The minor activity could be explained by forming a less active metabolite obtained from degradation or by reducing the guanidine derivative concentration inside the parasite and its activity. The formation of an active metabolite is confirmed for MAP2: in this case, the product of degradation was more active than the original compound in promastigote form. Similar results were found for GA-metabolite 5 obtained from the GA-Hecate peptide. Metabolite 5 (the degradation product) was the most efficient in inhibiting ZIKV strains of the African and Asian lineages during virus entry and replication [8].

One of the biggest global health problems is antimicrobial resistance, which causes around 9% of all global deaths. In 2019, approximately 13.7 million deaths were related to infectious diseases, of which those directly related to antimicrobial resistance accounted for around 1.27 million. The effects of morbidity or mortality related to antimicrobial resistance (AMR) are particularly severe in low- and middle-income countries, aggravating extreme poverty [35,36], causing a global economic problem. It is estimated that by 2050, low- and middle-income countries will have suffered an estimated loss of more than 5% of their gross domestic product because of these diseases [36,37]. In this manner, the current investment in developing new molecules for the treatment of infectious diseases contrasts sharply with an ever-increasing demand for new antimicrobials to treat infections caused by resistant microorganisms. In addition, the severity of leishmaniasis can be amplified by secondary infections of bacterial origin, whether opportunistic or pathogenic [38]. Studies using classic rodent models have advanced the understanding of how these bacteria interact with the host and disrupt its microbiota during the disease [39,40,41]. The development of new antileishmanial and bacterial compounds could solve this problem.

The peptides MAP1 and MAP2 showed activity against most bacterial strains. This behavior may be associated with the presence of lysine residues, which provided this peptide with a higher charge. Amphipathic peptides rich in lysine are multitargets for several pathogenic cells [24,42]. The addition of guanidine derivatives to peptides maintained the antimicrobial activity of the peptide. The GVL1-MAP2 bioconjugate generally exhibited similar or higher MIC values compared to GVL1-MAP1 against Gram-positive bacteria, indicating comparable or lower antibacterial potency. However, GVL1-MAP2 showed activity against all Gram-negative strains, whereas GVL1-MAP1 was active against fewer strains. Antimicrobial peptides typically have MICs in the 1–32 mg/mL range, as compared to the <1 mg/L used for traditional antibiotics [43]. In comparison to antimicrobial peptides that are already being used in clinical studies, such as the peptide nisin [44], the bioconjugate showed 8-fold less activity against the Gram-positive species *S. aureus*, but it showed the same activity against the Gram-negative species *P. aeruginosa* (different ATCC strain). These data showed the good activity of bioconjugates against bacteria with bactericidal effects in most cases.

The mechanism of action of peptides and bioconjugates was evaluated. Antimicrobial peptides (AMPs) can act on membranes, causing damage to them through mechanisms that lead to the formation of pores, such as barrel–stave, toroidal–pore, and transient pore, or causing the destabilization of the lipid bilayer, such as carpet-like mechanism [21,30]. The vesicle permeabilization studies showed that peptides and bioconjugates act in vesicles mimicking a *Leishmania* membrane, probably through carpet-like mechanism, with a rapid burst and instantaneous release of the fluorophore (Figure 7c,d). In this mechanism, the partially positively charged peptides adhere parallel to the membrane surface; the main binding force in this mechanism is assumed to be the electrostatic attraction between the negative charge of the vesicle bilayer surface that mimics the parasite membrane, from which micelles are formed [45]. For peptides in eukaryotic membrane–mimetic vesicles (Figure 7a,b), the leakage percentage increased with peptide concentration, characterized by a sustained release of a fluorophore. This kind of profile is attributed to the pore formation mechanism [46]. The difference in mechanisms in both vesicles is due to different lipid compositions. In addition, the conjugation strategy increases the capacity for membrane damage in eukaryotic mimetic vesicle (POPC/cholesterol). These data are in accordance with the CC_50_ found in macrophages, where the bioconjugates showed smaller values than those found for the peptides.

In addition to the membrane, the peptides may have other therapeutic targets. In concentrations lower than MIC, the peptide and bioconjugate may induce small and transient pores in the target membrane [47]. When the transient pores are broken down, the peptide sequence could cross the membrane and act on intracellular targets, as enzymes. Enzymes are essential for functional integrity and virulence. Cysteine proteases B (CPB) are important in the infectious process; their inhibition has shown high therapeutic potential, enabling the design of new drugs [48,49,50]. In *L. mexicana*, three cysteine protease genes were identified as virulence factors: *lmcpa*, *lmcpb* and *lmcpc*. The last encodes a cathepsin L-like protein and is the most abundant in the amastigote form of the parasite, making this enzyme an important therapeutic target. Guanidine derivatives target the cysteine protease, such as the compound E64 (L-trans-epoxysuccinyl-leucylamido-(4-guanidino)butane), which is a potent irreversible inhibitor of *Leishmania* CPB [51]. The guanidine derivative LQOF-G6 showed selective inhibitory activity on *Leishmania major* cysteine protease LmCPB2.8DCTE (CPB) and was not toxic [52]. Compounds belonging to the azapeptide class have also been reported to be commercial inhibitors of cysteine proteases, such as papain, cathepsin B, and cathepsin K. The peptide Z-Arg-Leu-Val-Gly-Ile-Val-Ome is also a potent inhibitor of cathepsin B, demonstrating the high potential of peptides as cysteine protease inhibitors [53].

The MAP2 peptide and the GVL1-MAP2 bioconjugate inhibited CPB enzyme activity. The enzymatic inhibition of the peptide was greater than that of the bioconjugate and can be explained by the increased size and volume of the molecule, which may cause steric hindrance in contact with the CPB enzyme site. GVL1 alone was also tested and showed the worst inhibitory action, indicating the peptide contribution in enzyme-bound areas. Cysteine proteases may also interfere with the parasite infection process. *Leishmania* expresses high levels of several classes of CP belonging to the papain family, which are crucial for the parasite’s metabolism, reproduction, and intracellular survival [54,55,56]. During the process of *Leishmania* infection in macrophages, CPB modulates the host’s responses by negatively regulating protective Th1 immune responses, particularly IFN-γ production, through degradation of the transcription factor NF-κB and subsequent inhibition of IL-12 production by infected macrophages. Therefore, the protozoan cysteine protease CPB can alter host macrophage signaling by increasing IL-12 transcription, which hampers the establishment of infection [49,57]. The literature demonstrates the fundamental role of parasite CPB in the infectious process and suggests the high therapeutic potential of inhibiting these enzymes [48,49,58,59]. Nonetheless, the bioconjugate showed a lower infection index than the MAP2 peptide. The bioconjugate could act on targets other than CPB, such as trypanothione reductase [7]. This could explain the higher activity of the bioconjugate.

These data show that GVL1-MAP2 is a multifunctional therapeutic tool. This bioconjugate not only overcomes the limitations of traditional AMPs regarding toxicity and stability [12,23] but also provides a protective immunomodulatory response essential for controlling *Leishmania* infection [51]. Together, the data obtained showed that the GVL1-MAP2 bioconjugate fulfills key pharmacological criteria for anti-*Leishmania* candidates, showing high potency and a selectivity index (SI > 10) that exceeds the requirements for visceral leishmaniasis treatment [16,18].

## 4. Materials and Methods

### 4.1. Peptides and Bioconjugates Synthesis

The synthesis of the peptides and their bioconjugates was made by the SPPS [13] using the Fmoc chemistry protocol [13]. The resin employed was the Rink amide-MBHAR resin (AAPPTEC, Louisville, KY, USA), which makes peptides with an amide at the C-terminal position.

The amino acid sequences that correspond to the MAP1 and MAP2 peptides are AAGKVLKLLKKLL-NH_2_ and AAKKVLKLLKKLL-NH_2_, respectively.

Their bioconjugates have GVL1 at the N-terminus. Coupling agents such as N,N’-diisopropylcarbodiimide (DIC)/1-hydroxybenzotriazole (HOBt) (Oakwood Chemical, Estill, SC, USA) or 2-(1-H-7-azabenzotriazol1-yl)-1,1,3,3-tetramethyluroniumhexafluorophosphate (HATU) (AAPPTEC, Louisville, KY, USA)/N,N-diisopropylethylamine (DIPEA) (Merck, Darmstadt, Germany), in N,N-dimethylformamide (DMF) (Êxodo Científica, Sumaré, SP, Brazil), are used for peptide bonds between Fmoc-amino acids (AAPPTEC, Louisville, KY, USA) and for the bioconjugation of the guanidine derivative moiety, respectively.

For the cleavage of the peptides and bioconjugates, a cocktail containing 2.5% H_2_O, 2.5% triisopropylsilane (TIS), and 95% trifluoroacetic acid (Oakwood Chemical, Estill, SC, USA) was used under constant agitation. After two hours, the peptides were precipitated with anhydrous ethyl ether (Êxodo Científica, Sumaré, SP, Brazil) and separated from the soluble non-peptide material by centrifugation. The peptide obtained was dissolved in an aqueous solution containing 0.045% TFA in water and lyophilized for subsequent characterization.

### 4.2. Molecule Purification and Characterization

The molecules were purified by reversed-phase high-performance liquid chromatography (HPLC), in semi-preparative mode using a C18 Sepax column (25 cm × 1 mm and 5 μm particle size) [7]. The molecules were then analyzed by mass spectrometry and HPLC to confirm their molecular weights and purity [7].

### 4.3. Antileishmanial Activity

*L. amazonensis* promastigotes (MPRO/BR/1972/M1841-LV-79) were cultivated in LIT medium, while *L. infantum* promastigotes (MNYC/BZ/62/M379) were raised in 199 culture medium (Sigma-Aldrich, St. Louis, MO, USA) supplemented with 10% heat-inactivated fetal bovine serum (FBS; Gibco/Invitrogen, Thermo Fisher Scientific, Waltham, MA, USA) [7] and 10% urine obtained from healthy male human donors among the members of the research group, at 28 °C, until the mid-log phase of growth was achieved. For macrophage recruitment, 2–3 mL of 3% thioglycolate was applied to the peritoneal cavity of the animals, three days before the start of the experiment. After this period, the animals were euthanized with CO_2_, and the macrophages were collected by washing the peritoneal cavity with 5 mL of sterile PBS. Viable cell counting was performed using trypan blue, and the concentration was adjusted to 1 to 5 × 10^6^ cells/mL. In a 96-well plate, 100 µL of the macrophage suspension was distributed per well and incubated for 4 to 6 h at 37 °C with 5% CO_2_ to allow for cell adhesion. After adhesion, the medium was removed, and 97 µL of complete RPMI medium and 3 µL of the compound to be tested were added, followed by incubation for 24 h, according to the methodology described by Almeida et al. [60]. For antileishmanial assays performed on the promastigote form to obtain IC_50_ measurements, cultures of the protozoans *L. infantum* and *L. amazonensis* were grown until the exponential phase and then transferred to 96-well plates at a final concentration of 1 × 10^7^ promastigotes × mL^−1^. Thereafter, the compounds of interest were added to those wells at concentrations ranging from 1.28 to 100 µmol L^−1^ to determine their anti-promastigote activity. After 72 h, viable protozoans were determined using the MTT method [61]. For antileishmanial assay performance on the amastigote form, macrophages were infected with *Leishmania infantum* and *Leishmania amazonensis* stationary-growth-phase promastigotes, using a ratio of five parasites per macrophage for *Leishmania amazonensis* and ten to one for *Leishmania infantum* at 37 °C in five-percent carbon dioxide for 4 h. Infected macrophages were treated with different concentrations of the peptides and bioconjugates and incubated for 24 h. The infection index was determined by the product of the percentage of infected macrophages and the average number of amastigotes per infected cell. All experiments were done in triplicate. The concentration responsible for inhibiting fifty percent of the growth of amastigote forms compared to the untreated control was determined by non-linear regression analysis using GraphPad Prism software. Normality was assessed using Shapiro–Wilk tests and statistical analysis (including ANOVA and the *t*-test), comparing the results with control groups and standard drugs, such as amphotericin B. Twelve healthy male Swiss mice obtained from the Central Vivarium of the School of Pharmaceutical Sciences—UNESP Araraquara (six to eight weeks of age, twenty-five to thirty grams) were used, eleven for the amastigote assay and one for the control. The infection indices were conducted as previously described [62].

### 4.4. Active Serum Stability

Peptide stability was investigated using anti-promastigote assays with *L. amazonensis* and *L. infantum* using LIT or 199 culture media with active fetal bovine serum, as previously detailed by de Souza et al. [7], in which the peptide is submitted to the same protocol as previously described for anti-promastigote assays, varying only the serum utilized: in this assay, the serum contains various proteases that can degrade the peptide, so it allows for a measurement of its stability when facing such enzymes.

### 4.5. CPB Enzyme Inhibition

CPB inhibition experiments were performed as described previously by Moreira et al., 2022 [52], utilizing pre-established methods consisting of the measurement of fluorescence, obtained from the cleavage of the substrate caused by the enzyme treated with the compounds.

### 4.6. Antibacterial Activity

The minimum inhibitory concentrations (MICs) of GVL, MAP1, GVL1-MAP1, MAP2 and GVL1-MAP2 were determined against clinically important species strains, including ESKAPE species: *S. epidermidis* ATCC35984, *E. faecalis* ATCC29212, *S. aureus* ATCC25923, *S. aureus* ATCC8095, *E. faecium* ATCC 700221, *K. pneumoniae* ATCC 700603, *E. coli* ATCC25922, *A. baumannii* ATCC19606, and *P. aeruginosa* ATCC27853. Although *S. epidermidis* is not part of the ESKAPE group, it was included due to its clinical relevance as an opportunistic pathogen associated with nosocomial infections. *S. epidermidis* is the most common cause of implant-associated infections and is a canonical opportunistic biofilm former. Each compound was diluted in water to prepare a 10× concentrated stock solution. Subsequently, the stock solution was diluted 1:10 in Cation-Adjusted Mueller Hinton broth (MHCA) (BD), according to CLSI (2013) [63]. Each compound was tested at 512 mg/mL or at the highest concentration at which it could be dissolved without precipitation. Serial dilutions (1:2) were made from the 512 mg/mL well to the 0.06 mg/mL well. Incubation was performed at 37 °C, and the results were visually read after 24 h, at which point the concentration at which the compound inhibited the microorganism’s growth was observed. For the negative and positive controls, MHCA broth was added. In the positive control, bacteria were added without the compound to observe their growth in MHCA broth. In the negative control, only the culture medium, MHCA broth, was present, without bacteria, to show that it was not contaminated. The tests were performed in triplicate.

To determine the minimum bactericidal concentration (MBC), 100 μL from each well corresponding to all tested concentrations in the MIC assay was subcultured onto MHCA agar plates and incubated at 35 °C for 24 h. The MBC was then determined as the lowest peptide concentration at which no visible growth was observed on the plate.

### 4.7. LUV Permeabilization Model Assay

Vesicles of POPC/cholesterol (4:1, m:m), for simulating human membranes, and POPG/POPS/ergosterol (8:1:1; m/m/m), for protozoan membrane simulation, containing fluorescein in their insides, were made according to the protocol described by Lorenzón et al. [64]. These vesicles had a carboxyfluorescein (CF) inner structure to detect extravasation via an increase in fluorescence due to pore formation. The wavelength of excitation used was 490 nm, and the wavelength of emission was 512 nm.

For a blank passage, the vesicles were introduced to the spectrofluorometer in a quartz cuvette, diluted in PBS, for a time of 480 s (0% of release). Then, a 10% solution of Triton (polyethylene glycol p-(1,1,3,3-tetramethylbutyl)-phenyl ether) was added to the cuvette, therefore releasing all the fluorescent material (100% of release). Evaluation of the GVL1, peptide, and bioconjugate in concentrations of 50 µM and 10 µM was done by the addition of these compounds at 120 s. After 480 s, Triton was added to reach the maximum read of fluorescence (100% of release).

### 4.8. Cytotoxicity Assay on Murine Peritoneal Macrophages

Macrophages were sourced from the peritoneal cavity of Swiss mice (details in Section 4.3), using the methodology outlined by Almeida et al., 2017 [65]. The MTT colorimetric assay was carried out. The drug concentration corresponding to 50% cell growth inhibition was expressed as the cytotoxic concentration (CC_50_). Seven male Swiss mice (six to eight weeks of age; twenty-five to thirty grams) were used, six for the cytotoxicity assay and one for the control.

### 4.9. Statistical Analysis

Data were expressed as the mean ± standard deviation (SD) of at least three independent experiments performed in triplicate. The normality of the data distribution was assessed using the Shapiro–Wilk test. For comparisons between multiple groups, such as the antileishmanial and antibacterial assays, one-way analysis of variance (ANOVA), followed by Tukey’s post hoc test, was employed. For enzyme inhibition assays, comparisons were made using Student’s *t*-test.

The IC_50_, CC_50_, and EC_50_ values were determined by non-linear regression analysis (log[inhibitor] vs. response—variable slope). All statistical analyses and graphical representations were performed using GraphPad Prism software version 8.0 (GraphPad Software, San Diego, CA, USA). A *p*-value of less than 0.05 (*p* < 0.0) was considered “statistically significant.”

All assays were performed in triplicate.

## 5. Conclusions

This study describes the successful development of bioconjugate containing guanidine derivatives and antimicrobial peptides as an effective strategy for creating new compounds to treat leishmaniasis and bacterial infections. The key achievement is obtaining a compound with high antileishmanial activity and elevated selectivity, surpassing the lack of selectivity observed with amphotericin B, the current standard treatment. In addition, the development of antibacterial and antileishmanial compounds can avoid secondary infections, whether opportunistic or pathogenic. The bioconjugation strategy proved effective in enhancing the compound’s potency and selectivity, offering a superior alternative for developing new drugs. Although degradation assays indicated that the bioconjugate suffers degradation in active serum, it nonetheless maintained its biological activity, suggesting sufficient functional stability. Permeabilization studies and enzyme inhibition analyses revealed that the peptide acts via at least two distinct mechanisms, with the primary one focusing on the membrane and inner targets as an inhibitor of CPB enzyme. These results provide valuable information for designing new antileishmanial and antibacterial peptides. The use of the guanidine derivative–peptide conjugation shows excellent potential in new drug discovery, offering crucial insights for the development of therapeutics against leishmaniasis and bacteria, significant global public health concerns.

## Figures and Tables

**Table 1:** Antileishmanial activity of MAP1, MAP2, GVL1, GVL1-MAP1, GVL1-MAP2 and Amp B against *L. amazonensis.* The assays were performed in triplicate, in three events.

Compound	IC_50_ (µM) Promastigote	IC_50_ (µM) Amastigote	CC_50_ (µM) Macrophage	SI (Amastigote)
GVL1	51.8 ± 0.10	7.5 ± 3.50	>2000 ± 0.01	>267
MAP1	43.0 ± 4.20	10.0 ± 1.24	117 ± 1.20	12
GVL1-MAP1	7.3 ± 2.70	0.9 ± 0.05	21 ± 2.80	23
MAP2	NA	5.0 ± 1.00	135 ± 0.02	27
GVL1-MAP2	3.2 ± 0.50	0.6 ± 0.10	125 ± 0.05	201
Amp B	3.3 ± 0.20	0.75 ± 0.10	24 ± 0.02	32

NA: The compound showed no activity at the maximum concentration tested (100 µM).

**Table 2:** Antileishmanial activity of GVL1, MAP2, GVL1-MAP2 and Amp B against *L. infantum*. The assays were performed in triplicate, in three events.

Compound	IC_50_ (µM) Promastigote	IC_50_ (µM) Amastigote	CC_50_ (µM) Macrophage	SI (Amastigote)
MAP2	NA	7.80 ± 0.50	135 ± 0.02	17
GVL1-MAP2	2.50 ± 0.50	1.00 ± 0.40	125 ± 0.05	125
GVL1	50.0 ± 2.20	9.50 ± 2.50	>2000 ± 0.01	>210
Amp B	5.40 ± 0.50	1.25 ± 0.10	24 ± 0.02	19

NA: The compound showed no activity at the maximum concentration tested (100 µM).

**Table 3:** IC_50_ values (concentration capable of killing 50% of the parasites) obtained in anti-promastigote assays with *L. amazonensis* and *L. infantum* in active serum. The assays were performed in triplicate, in three events.

Compound	IC_50_ (µM) *L. amazonensis*	IC_50_ (µM) *L. infantum*
GVL1-MAP2	9.9 ± 0.20	4.4 ± 0.80
MAP2	81.5 ± 1.40	17.4 ± 5.40

**Table 4:** MIC, MBC, and antibacterial activity of GVL1, MAP1, and GVL1-MAP1 against Gram-positive and Gram-negative bacteria (highlighted in gray). Values are expressed in mg/mL. The assays were performed in triplicate, in three events.

Bacterial Strains	MIC (mg/mL)			MBC (mg/mL)			MBC/MIC		Activity *	
	GVL1	MAP1	GVL1-MAP1	GVL1	MAP1	GVL1-MAP1	MAP1	GVL1-MAP1	MAP1	GVL1-MAP1
*S. epidermidis* ATCC 35984	128	4	16	>512	8	32	2	2	Bactericide	Bactericide
*S. aureus* ATCC 25923	256	128	16	512	128	32	1	2	Bactericide	Bactericide
*S. aureus* ATCC 8095	128	64	16	>512	64	32	1	2	Bactericide	Bactericide
*E. faecalis* ATCC 29212	256	512	64	>512	>512	512	N.D.	8	N.D.	Bacteriostatic
*E. faecium* ATCC 700221	256	16	8	>512	64	16	4	2	Bactericide	Bactericide
*K. pneumoniae* ATCC 700603	>512	16	>256	N.R.	16	N.R.	1	N.R.	Bactericide	N.R.
*E. coli* ATCC 25922	>512	16	>256	N.R.	16	N.R.	1	N.R.	Bactericide	N.R.
*A. baumannii* ATCC 19606	>512	16	32	N.R.	16	32	1	1	Bactericide	Bactericide
*P. aeruginosa* ATCC 27853	>512	32	64	N.R.	128	>256	4	N.D.	Bactericide	N.D.

* Activity defined by the MBC/MIC ratio. For results = 4 or <4, the activity is considered bactericidal; above this value, the activity is considered bacteriostatic, according to Pankey and Sabath (2004) [14]. Undetermined activity (N.D.). Test not performed as the compound did not show activity against the strain (N.R.).

**Table 5:** MIC, MBC, and antibacterial activity of MAP2 and GVL1-MAP2 against Gram-positive and Gram-negative bacteria (highlighted in gray). Values are expressed in mg/mL. The assays were performed in triplicate, in three events.

Bacterial Strains	MIC (mg/mL)		MBC (mg/mL)		MBC/MIC		Activity *	
	MAP2	GVL1-MAP2	MAP2	GVL1-MAP2	MAP2	GVL1-MAP2	MAP2	GVL1-MAP2
*S. epidermidis* ATCC 35984	16	32	16	64	1	2	Bactericide	Bactericide
*S. aureus* ATCC 25923	128	32	256	32	2	1	Bactericide	Bactericide
*S. aureus* ATCC 8095	32	16	64	16	2	1	Bactericide	Bactericide
*E. faecalis* ATCC 29212	512	64	512	64	1	1	Bactericide	Bactericide
*E. faecium* ATCC 700221	32	16	64	16	2	1	Bactericide	Bactericide
*K. pneumoniae* ATCC 700603	32	64	32	64	1	1	Bactericide	Bactericide
*E. coli* ATCC 25922	32	64	32	64	1	1	Bactericide	Bactericide
*A. baumannii* ATCC 19606	16	32	32	32	2	1	Bactericide	Bactericide
*P. aeruginosa* ATCC 27853	128	64	256	64	2	1	Bactericide	Bactericide

* Activity defined by the MBC/MIC ratio. For results = 4 or <4, the activity is considered bactericidal; above this value, the activity is considered bacteriostatic, according to Pankey and Sabath (2004) [14]. Undetermined activity (N.D.). N.R.: Test not performed as the compound did not show activity against the strain.

**Table 6:** Results for CPB enzyme inhibition. The assays were performed in triplicate, in three events.

Compound	CPB (20 μM) Inhibitory Activity (%)	IC_50_ CPB Inhibitory Activity (μM)
MAP2	94.60 ± 0.26	1.50 ± 0.20
GVL1-MAP2	88.90 ± 2.12	3.80 ± 0.30
GVL1	14 ± 2.80	---

## Data Availability

The original contributions presented in this study are included in the article/supplementary material. Further inquiries can be directed to the corresponding authors.

## Supplementary Materials

The following supporting information can be downloaded at https://www.mdpi.com/article/10.3390/ph19060835/s1: Figure S1. Structure of the guanidine derivative GVL1. Figure S2. Structure of the peptide MAP1 (AAGKVLKLLKKLL-NH_2_). Figure S3. Structure of the peptide MAP2 (AAKKVLKLLKKLL-NH_2_). Figure S4. Mass spectrum of the MAP1 peptide. Figure S5. Mass spectrum of the MAP2 peptide.

## Abbreviations

The following abbreviations are used in this manuscript:

AMPs	Antimicrobial peptides
Amp B	Amphotericin B
AMR	Antimicrobial resistance
CF	Carboxyfluorescein
CL	Cutaneous leishmaniasis
CPB	Cysteine proteases B
CPP	Cell penetration peptide
DIEA	N,N-Diisopropylethylamine
GIPL	Glycoinositolphospholipids
GVL1	(Z)-4-((2-benzoyl-3-(4-bromophenyl)guanidino)methyl) benzoic acid
HATU	2-(1-H-7-azabenzotriazol1-yl)-1,1,3,3-tetramethyluroniumhexafluorophophate
LPG	Lipophosphoglycan
MAP1	AAGKVLKLLKKLL-NH_2_
MAP2	AAKKVLKLLKKLL-NH_2_
MBC	Minimum bactericidal concentration
MIC	Minimum inhibitory concentration
POPC	1-palmitoyl-2-oleoyl-sn-glycero-3-phosphocholine
POPS	1-palmitoyl-2-oleoyl-sn-glycero-3-phospho-L-serine
SI	Selectivity index (MBC/MIC ratio)
