# Supplementary material for: Evaluation of Antileishmanial and Antibacterial Activity of Bioconjugate Guanidine–Temporin
Source: Pharmaceuticals (Basel). 2026 May 27;19(6):835. doi: 10.3390/ph19060835 (PMC13305352; doi:10.3390/ph19060835)
Supplement: Supplementary file 1 [file pharmaceuticals-19-00835-s001.zip › pharmaceuticals-4286990-supplementary.pdf]

Supporting Information

Evaluation of Antileishmanial and Antibacterial Activity of Bioconjugate Guanidine–Temporin

Gabriel Antunes Santoro [1,†], Natalia Caroline Souza Costa [2,†], Sarah Tolentino Rocha Brandão [1], Jhonatan Santos de Lima [2], Angela Maria Arenas Velasquez [2], Luana Ribeiro dos Anjos [3], Cauã Dias Abrão [3], João Victor Marcelino de Souza [1], Marcela Nunes Argentin [4], Ilana Lopes Baratella da Cunha Camargo [4], Marcia A. S. Graminha [2], Eduardo Rene P. Gonzalez [3,*] and Eduardo Maffud Cilli [1,*]

[1] Department of Biochemistry and Organic Chemistry, Institute of Chemistry, São Paulo State University (UNESP), Araraquara 14800-060, SP, Brazil; ga.santoro@unesp.br (G.A.S.); sarah.brandao@unesp.br (S.T.R.B.); joao-victor.souza@unesp.br (J.V.M.d.S.)
[2] School of Pharmaceutical Sciences, São Paulo State University (UNESP), Araraquara 14800-903, SP, Brazil; natalia.costa@unesp.br (N.C.S.C.); jhonatan.lima@unesp.br (J.S.d.L.); a.velasquez@unesp.br (A.M.A.V.); marcia.graminha@unesp.br (M.A.S.G.)
[3] School of Sciences and Technology, São Paulo State University (UNESP), Presidente Prudente 19060-080, SP, Brazil; luana.anjos@unesp.br (L.R.d.A.); dias.abrao@unesp.br (C.D.A.)
[4] Department of Physics and Interdisciplinary Science, São Carlos Institute of Physics, University of São Paulo (USP), São Carlos 13563-120, SP, Brazil; marcela.argentin@usp.br (M.N.A.); ilanacamargo@ifsc.usp.br (I.L.B.d.C.C.)
* Correspondence: eduardo.gonzalez@unesp.br (E.R.P.G.); eduardo.cilli@unesp.br (E.M.C.)
† These authors contributed equally to this work.

Table of Contents:

Supporting Figures

Figure S1. Structure of the guanidine derivative GVL1…………………………………..S2
Figure S2. Structure of the peptide MAP1 (AAGKVLKLLKKLL-NH_2)………….…….S2
Figure S3. Structure of the peptide MAP2 (AAKKVLKLLKKLL-NH_2) ……….………..S2
Figure S4. Mass spectrum of the MAP1 peptide ……….……………………….……....S3
Figure S5. Mass spectrum of the MAP2 peptide ………….………..………….……….S4

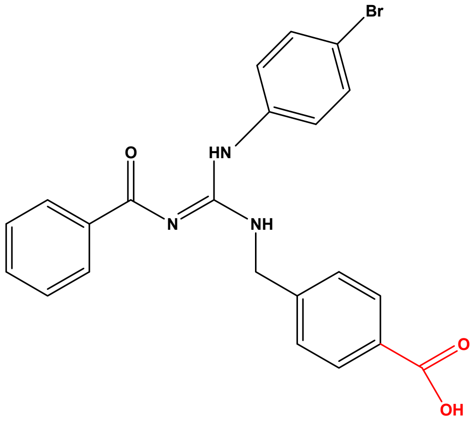

Figure S1. Structure of the guanidine derivative GVL1. The carboxylic group used in peptide conjugation is red.

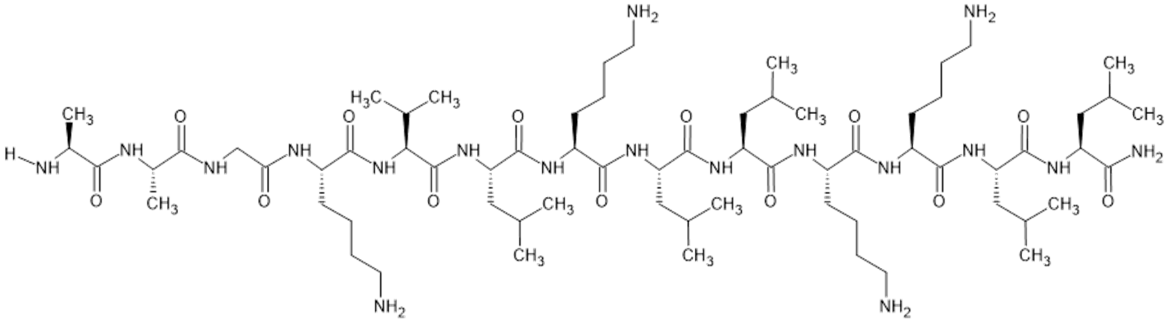

Figure S2. Structure of the peptide MAP1 (AAGKVLKLLKKLL-NH_2).

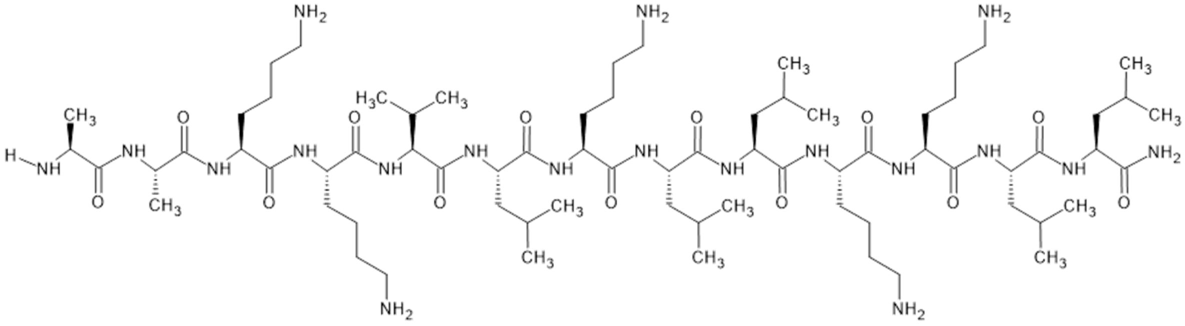

Figure S3. Structure of the peptide MAP2 (AAKKVLKLLKKLL-NH_2).

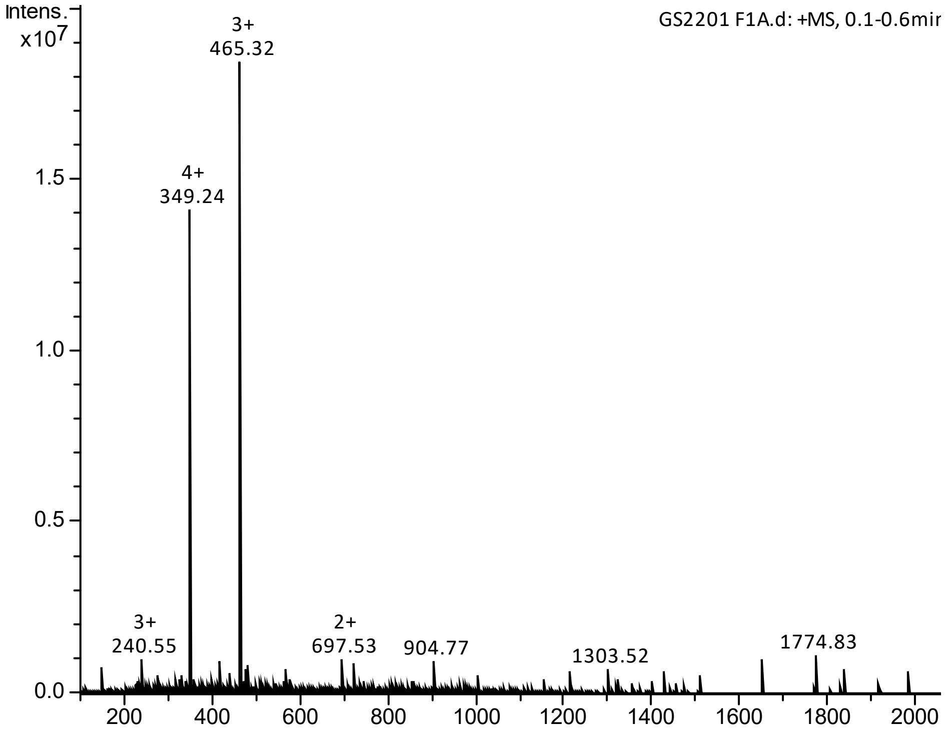

Figure S4. Mass spectrum of the MAP1 peptide. Thermo LCQ-fleet mass spectrometer, with ESI-IT-MS configuration. Direct infusion of the sample solutions was carried out at a concentration of about 10 ppm in acetonitrile/water containing 0.1% v/v formic acid. The infusion flow was adjusted to 5.0 µL/min and the electrospray source was operated in positive mode, applying 4.5 kV to the electrospray capillary. Obtained MW/Z = 697.52 (Z=2); 465.32 (Z=3) and 349.22 (Z=4). Theorical MW = 1393,85 g mol^{-1}.

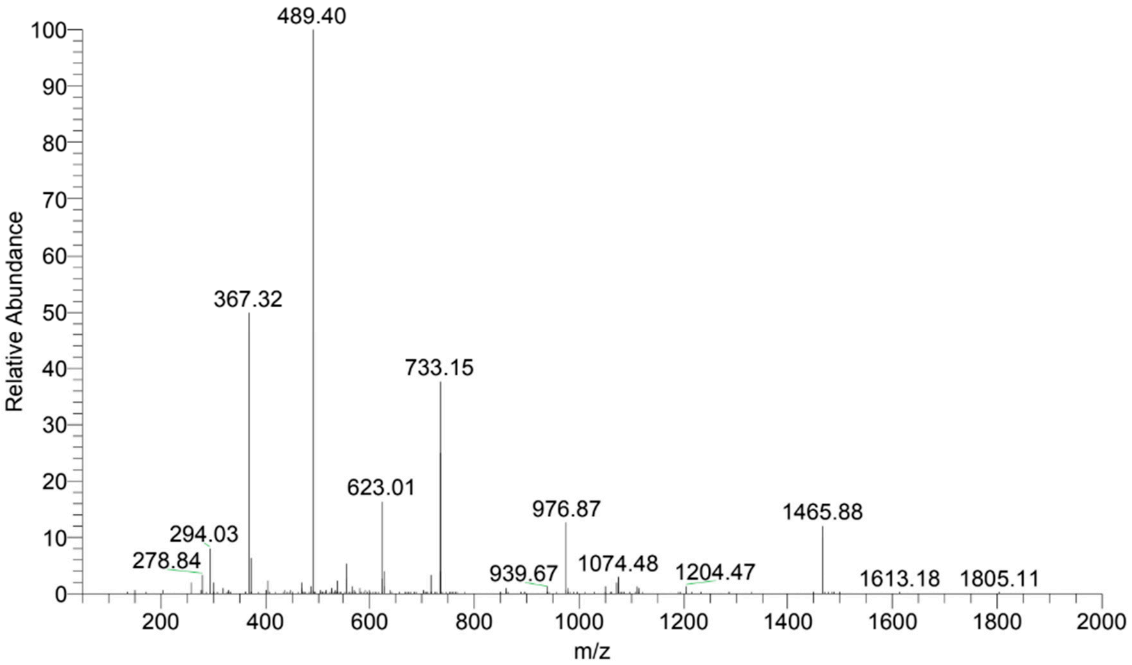

Figure S5. Mass spectrum of the MAP2 peptide. Thermo LCQ-fleet mass spectrometer, with ESI-IT-MS configuration. Direct infusion of the sample solutions was carried out at a concentration of about 10 ppm in acetonitrile/water containing 0.1% v/v formic acid. The infusion flow was adjusted to 5.0 µL/min and the electrospray source was operated in positive mode, applying 4.5 kV to the electrospray capillary. Obtained MW/Z = 1465.88 (Z=1); 733.15 (Z=2) and 489,40 (Z=3). Theorical MW = 1464,97 g mol^{-1}.
